# Prevalence and Associated Factors for Pterygium in Rural Agrarian Central India. The Central India Eye and Medical Study

**DOI:** 10.1371/journal.pone.0082439

**Published:** 2013-12-04

**Authors:** Vinay Nangia, Jost B. Jonas, Deepa Nair, Nandita Saini, Prabhat Nangia, Songhomitra Panda-Jonas

**Affiliations:** 1 Suraj Eye Institute, Nagpur, Maharashtra, India; 2 Department of Ophthalmology, Medical Faculty Mannheim of the Ruprecht-Karls-University, Heidelberg, Mannheim, Germany; Zhongshan Ophthalmic Center, China

## Abstract

**Purpose:**

To evaluate the prevalence of pterygia and associated factors in a rural population in a mostly undeveloped agrarian region.

**Methods:**

The Central India Eye and Medical Study is a population-based study performed in a rural region of Central India. The study comprised 4711 subjects (aged 30+ years). A detailed ophthalmic and medical examination was performed. A pterygium was diagnosed during the slit lamp examination and confirmed on corneal photographs. It was defined as a fleshy fibrovascular growth, crossing the limbus and typically seen on the nasal, and sometimes temporal, conjunctiva.

**Results:**

A pterygium was detected in 798 eyes (prevalence rate: 8.47±0.29%) of 608 (12.91±0.49%) subjects. Bilateral pterygia were present in 190 subjects (4.0% of study population). Pterygia prevalence increased from 6.7±0.8% in the age group 30-39 years, to 13.5±1.2% in the age group 50-59 years, to 25.3±2.1% in the age group 70-79 years. Prevalence of pterygia was associated with older age (*P*<0.001; regression coefficient B: 0.02; odds ratio (OR): 1.02; 95%CI: 1.01, 1.03), male gender (*P*<0.001;B:-0.73;OR: .48;95%CI:0.39,0.61), lower level of education (*P*<0.001;B:-0.30;OR:0.74;95%CI:0.69,0.80), lower body height (*P*=0.001;B:-0.02;OR:0.98;95%CI:0.97,0.99), and higher cylindrical refractive error (*P*<0.001;B:0.23;OR:1.26;95%CI:1.18,1.34). If the education level was dropped, the number of hours spent with vigorous activity outdoors (*P*=0.001;B:0.001;OR:1.001;95%CI:1.000,1.0001) was significantly associated with the prevalence of pterygia, in addition to older age (*P*<0.001;B:0.03;OR:1.03;95%CI:1.03,1.04), male gender (*P*<0.001;B:-0.49;OR:0.62;95%CI:0.49,0.77), lower body height (*P*=0.005;B:-0.02;OR:0.98;95%CI:0.97,0.99), and higher cylindrical refractive error (*P*<0.001;B:0.23;OR:1.25;95%CI:1.18,1.34).

**Conclusions:**

Pterygium prevalence in rural Central India is about 13% among adult Indians aged 30+ years. Older age, male gender, lower educational level, lower body height and more time spent outdoors with vigorous work were associated factors. Since the living conditions in the study location were mostly untouched by modern developments, the results may show the prevalence and associations of pterygia without major medical or technologic influences.

## Introduction

Pterygium is a wedge-shaped fibrovascular dysplasia of the bulbar conjunctiva and is usually located in the nasal horizontal, or more rarely, in the temporal horizontal part of the limbus. Its prevalence was examined in numerous previous population-based studies which suggested that countries closer to the equator had a higher prevalence due to higher levels of outdoors ultraviolet radiation exposure [1,32]. The potential role of other causal mechanisms and risk factors as predisposition to the disease have mostly remained elusive so far. Since the previous studies were carried out in regions with a minimum of technological and socioeconomic development, we examined the prevalence of pterygia in a population which has mostly been bereft of major developments yet, where lifestyle and living conditions have not markedly changed within the last 100 years, and in an area which lacks medical infrastructure and therefore interference by the treatment of other diseases is unlikely. 

## Methods

### Ethics Statement

The Medical Ethics Committee of the Medical Faculty Mannheim of the Ruprecht-Karls-University Heidelberg and a similar committee of the Suraj Eye Institute/Nagpur approved the study; all participants gave informed written consent, according to the Declaration of Helsinki. 

The Central India Eye and Medical Study (CIEMS) is a population-based cross-sectional study in Central India. The study was carried out in 8 villages in the rural region of Central Maharashtra at a distance of about 40 km from Nagpur [33,34]. The villages were at the border of tribal regions. Inclusion criterion was an age of 30+ years. Out of 5885 eligible subjects, 4711 (80.1%) people (2520 (53.5%) women) participated. The examinations were carried out in the hospital. Trained social workers filled out a questionnaire with 200 items on the socioeconomic background, living conditions, daily food, smoking or other types of tobacco consumption, alcohol consumption, daily physical activity, known diagnosis of major systemic diseases, intake of medication, psychiatric status (questions on psychiatric depression including thoughts of suicide), family history of eye diseases, and wearing and availability of glasses. The level of education was graded into 5 stages, with “0” for “illiterate”, “1” for “1 to 5th grade”, “2” for “6 to 8th grade”, “3” for “9th to 12th grade”, and “4” for “college”. The number of hours spent with vigorous work outdoors was noted. 

As an assessment tool for smoking behavior the Fagerstrom Test for Nicotine Dependence (FTND) was used [35]. The FTND is an adaptation of the original Fagerstrom Nicotine Tolerance Questionnaire with six simple questions to determine the level of current nicotine addiction. In addition to the FTND, the history of former nicotine consumption (age of onset of tobacco consumption, cessation age, estimated pack years), consumption of different tobacco products (hookah, bidis, smokeless tobacco products like Snuff), and chewing tobacco and betel nut was recorded by 9 additional standardized questions. To determine alcohol consumption, the Alcohol Use Disorders Identification Test (AUDIT) was utilized [36]. The Audit is a 10-item self report questionnaire in which a sum score of 8 or more is associated with harmful or hazardous drinking, and in which a score of 13 or more in women, and 15 or more in men, is likely to indicate alcohol dependence. In addition to the AUDIT, the history of former alcohol consumption (starting age of alcohol consumption, cessation age) and the current drinking behavior (e. g. type of drink) was assessed by additional 4 standardized questions.

The ophthalmic examinations included assessment of presenting, uncorrected and best corrected visual acuity, keratometry (non-automatic keratometer; Appassawamy Ass., Chennai, India), frequency-doubling perimetry (program C-20-1; Zeiss-Humphrey, Dublin, California, USA), corneal photography, slit lamp assisted biomicroscopy of the anterior segment was noted, Goldmann applanation tonometry, corneal pachymetry, ocular biometry (Pacscan; Sonomed, U.S.A), and after induction of medical mydriasis, digital photography of the lens, optic disc and macula (ZeisFF450 telecentric fundus camera; Zeiss Meditec Co. Oberkochen, Germany). Using the corneal photographs, corneal arcus was semi-quantified in a masked fashion into 6 grades with “0” for “no corneal arcus”, and “5” for maximal corneal arcus”. A pterygium was diagnosed during the slit lamp examination of the cornea carried out by an experienced fellowship trained ophthalmologist and confirmed on the corneal photographs by a senior ophthalmologist (VN) It was defined as a fleshy fibrovascular growth, crossing the limbus and typically seen on the nasal conjunctiva. 

Statistical analysis was performed using a commercially available statistical software package (SPSS for Windows, version 21.0, IBM-SPSS, Chicago, IL). In a first step, we determined the mean prevalence of pterygia, presented as mean ± standard error. Other parameters were presented as mean ± standard deviation. In a second step, we performed univariate analyses of the associations between the prevalence of pterygia and other systemic parameters and ocular variables. In a third step, we carried out binary regression analyses with the prevalence of pterygia as the dependent parameter and with all those variables as independent parameters that were significantly associated with the prevalence of pterygia in the univariate analyses. Odds ratios (OR) and 95% confidence intervals (CI) were presented. A *P*-value <0.05 was considered to indicate statistical significance.

## Results

A pterygium was detected in 798 eyes (prevalence rate: 8.5 ± 0.3%; 95% CI: 7.9, 9.0) of 608 (12.9 ± 0.5%; 95%CI: 12.0, 13.9) subjects (Table 1). Bilateral pterygia were present in 190 subjects (31.3% of the subjects with pterygium or 4.0% of the whole study population). The subjects with pterygia were significantly older than the subjects without pterygia (56.6 ± 13.0 years (median: 60 years; range: 30 - 85 years) versus 48.8 ± 13.2 years (median: 45 years; range: 30 - 1000 years); *P*<0.001). Prevalence of pterygia increased from 6.7 ± 0.8% in the age group from 30-39 years, to 13. 5 ± 1.2% in the age group from 50-59 years, to 25.3 ± 2.1% in the age group from 70-79 years. Including only subjects with an age of 40+ years, the prevalence was 14.8 ± 0.6%; 95% CI: 13.7, 16.0). 

**Table 1:** Prevalence rates of pterygium in the population of the central India Eye and Medical Study stratified by age and gender.

	Men			Women			All		
Age (Years)	n (Total)	n (% ± Standard Error)	95% Confidence IntervaI	n (Total)	n (% ± Standard Error)	95% Confidence IntervaI	n (Total)	n (% ± Standard Error)	95% Confidence IntervaI
30-39	490	35 (7.14 ± 1.17)	4.85, 9.43	630	40 (6.35 ± 0.97)	4.44, 8.26	1120	75 (6.70 ± 0.75)	5.23, 8.16
40-49	632	63 (9.97 ± 1.19)	7.63, 12.31	742	49 (6.60 ± 0.91)	4.81, 8.39	1374	112 (8.15 ± 0.74)	6.70, 9.60
50-59	397	66 (16.62 ± 1.87)	12.95, 20,30	406	42 (10.34 ± 1.51)	7.37, 13.32	803	108 (13.45 ± 1.21)	11.08, 15.81
60-69	354	82 (23.16 ± 2.25)	18.75, 27.58	543	102 (18.78 ± 1.68)	15.49, 22.08	897	184 (20.51 ± 1.35)	17.87, 23.16
70-79	261	66 (25.29 ± 2.70)	19.98, 30.60	182	46 (25.27 ± 3.23)	18.90, 31.65	443	112 (25.28 ± 2.07)	21.22, 29.35
80+	57	15 (26.32 ± 5.88)	14.53, 38.10	17	2 (11.76 ± 8.06)	-5.31, 28.84	74	17 (23.97 ± 4.92)	13.16, 32.79
30+	2191	327 (14.92 ± 0.76)	13.43, 16.42	2520	281 (11.15 ± 0.63)	9.92, 12.38	4711	608 (12.91 ± 0.49)	11.95, 13.86

 The subjects with bilateral pterygium as compared with those with unilateral pterygium were significantly older (*P*=0.005) and had a lower level of education (*P*<0.001), while both groups did not vary significantly in gender (*P*=41), cylindrical refractive error (*P*=0.07), number of hours outdoors with vigorous work (*P*=0.17), and axial length (*P*=0.25). In a multivariate analysis, the presence of bilateral pterygia versus unilateral pterygium was significantly associated with male gender (*P*=0.006; regression coefficient B: -0.58; OR: 0.56; 95%CI: 0.37, 0.85), lower body height (*P*=0.049; B: -0.02; OR: 0.98; 95%CI: 0.96, 1.00), lower level of education (*P*<0.001; B: -0.28; OR: 0.75; 95%CI: 0.65, 0.87). If the level of education was dropped as independent parameter, the number of hours spent with vigorous outdoors activity 

 In univariate analysis, the overall presence of pterygia was significantly associated with the systemic parameters of older age (*P*<0.001), male gender (*P*<0.001), lower level of education (*P*<0.001), lower number of smoking cigarette package years (*P*=0.02), lower body height (*P*=0.002), lower body weight (*P*<0.001), lower body mass index (*P*=0.001), and higher systolic blood pressure (*P*<0.001), and the ocular parameters of higher cylindrical refractive error (*P*<0.001), lower best corrected visual acuity (*P*<0.001), more visual field defects (*P*=0.03), presence of pseudoexfoliation (*P*=0.005), degree of corneal arcus (*P*=0.01), lower intraocular pressure measurements (*P*=0.02), lower retinal nerve fiber layer cross section area (*P*<0.001), and higher prevalence of glaucoma (*P*=0.03) (Table 2). It was not associated with diastolic blood pressure (*P*=0.88), alcohol consumption (*P*=0.25), depression score (*P*=0.56), blood concentration of cholesterol (*P*=0.15), glycosylated hemoglobin (*P*=0.13), refractive error (*P*=0.12), axial length (*P*=0.80), anterior chamber depth (*P*=1.00), lens thickness (*P*=0.39), anterior corneal refractive power (*P*=0.79), central corneal thickness (*P*=1.00), optic disc area (*P*=0.49), neuroretinal rim area (*P*=0.46), and prevalence of early age-related macular degeneration (*P*=1.00), branch retinal vein occlusion (*P*=1.00), central retinal vein occlusion (*P*=1.00), diabetic retinopathy (*P*=0.24), disc hemorrhages (*P*=1.00), retinitis pigmentosa (*P*=0.59) and myopic retinopathy (*P*=0.30). 

**Table 2:** Associations (univariate analysis) between the presence of pterygium and systemic or ocular parameters in the Central India Eye and Medical Study.

Parameter	*P*-Value	Regression Coefficient B	Odds Ratio	95% Confidence Interval of Odds Ratio
Age (Years)	<0.001	0.04	1.04	1.04, 1.05
Men / Women	<0.001		0.71	0.62, 0.82
Level of Education (1-5)	<0.001	-0.40	0.67	0.63, 0.72
Package Years of Smoking	0.02	0.009	1.01	1.00, 1.02
Body Height (cm)	0.002	-0.01	0.99	0.98, 1.00
Body Weight (kg)	<0.001	-0.02	0.98	0.98, 0.99
Body Mass Index (kg/m^2^)	0.001	-0.04	0.96	0.94, 0.98
Systolic Blood Pressure (mm Hg)	<0.001	0.01	1.01	1.01, 1.01
Cylindrical Refractive Error	<0.001	0.39	1.48	1.37, 1.60
Best Corrected Visual Acuity (logMAR)	<0.001	0.62	1.86	1.60, 2.16
Visual Field Defects	0.03	0.02	1.02	1.00, 1.04
Presence of Pseudoexfoliation	0.005		2.34	1.38, 4.11
Degree of Corneal Arcus (0-5)	0.01	0.28	1.33	1.06, 1.66
Retinal Nerve Fiber Layer Cross Section Area	<0.001	-0.59	0.56	0.44, 0.71
Presence of Glaucoma	0.03		1.66	1.08, 2.57
Intraocular Pressure (mmHg)	0.02	-0.03	0.97	0.95, 1.00
Diastolic Blood Pressure (mmHg)	0.88			
Alcohol Consumption	0.25			
Depression Score	0.56			
Blood Concentration of Cholesterol	0.15			
HbA1c	0.13			
Refractive Error (Diopter)	0.12			
Axial Length (mm)	0.80			
Anterior Chamber Depth (mm)	1.00			
Lens Thickness (mm)	0.39			
Anterior Corneal Refractive Power (Diopter)	0.79			
Central Corneal Thickness (µm)	1.00			
Optic Disc Area (mm^2^)	0.49			
Neuroretinal Rim Area (mm^2^)	0.46			
Prevalence of Early Age-Related Macular Degeneration	1.00			
Prevalence of Branch Retinal Vein Occlusion	1.00			
Prevalence of Central Retinal Vein Occlusion	1.00			
Prevalence of Diabetic Retinopathy	0.24			
Presence of Disc Hemorrhage	1.00			
Prevalence of Retinitis Pigmentosa	0.59			
Prevalence of Myopic Retinopathy	0.30			

 Based on the results of the univariate analysis, we performed a binary regression analysis, with the presence of a pterygium as dependent variable and all those variables as independent parameters which were significantly associated with pterygia in the univariate analysis. In a step-wise manner, we then dropped those independent parameters which were no longer significantly associated with the presence of pterygia, starting with the parameters with the highest *P*-values. We dropped the presence of body weight (*P*=0.33), best corrected visual acuity (*P*=0.93), number of smoking cigarette package years (*P*=0.87), number of perimetric defects (*P*=0.67), presence of pseudoexfoliation (*P*=0.94), body mass index (*P*=0.67), intraocular pressure (*P*=0.58), hours spent with vigorous activity (*P*=0.52), presence of glaucoma (*P*=0.41), degree of corneal arcus (*P*=0.29), retinal nerve fiber layer cross sectional area (*P*=0.80), and systolic blood pressure (*P*=0.09). Finally, the prevalence of pterygia was associated with older age (*P*<0.001; B: 0.02; OR: 1.02; 95%CI: 1.01, 1.03), male gender (*P*<0.001; B: -0.73; OR: 0.48; 95%CI: 0.39, 0.61), lower level of education (*P*<0.001; B: -0.30; OR: 0.74; 95%CI: 0.69, 0.80), lower body height (*P*=0.001; ORB: -0.02; : 0.98; 95%CI: 0.97, 0.99), and higher cylindrical refractive error (*P*<0.001; B: 0.23; OR: 1.26; 95%CI: 1.18, 1.34). 

 If the level of education was dropped as independent parameter, the number of hours spent with vigorous outdoors activity (*P*=0.001; B: 0.001; OR: 1.001; 95%CI: 1.000, 1.0001) was significantly associated with the prevalence of pterygia, in addition to older age (*P*<0.001; B: 0.03; OR: 1.03; 95%CI: 1.03, 1.04), male gender (*P*<0.001; B: -0.49; OR: 0.62; 95%CI: 0.49, 0.77), lower body height (*P*=0.005; B: -0.02; OR: 0.98; 95%CI: 0.97, 0.99), and higher cylindrical refractive error (*P*<0.001; B: 0.23; OR: 1.25; 95%CI: 1.18, 1.34). 

## Discussion

In our population-based study in rural Central India, the prevalence rate of pterygia was 8.5 ± 0.3% per eye and 12.9 ± 0.5% per subject. Bilateral pterygia were present in 31.3% of the subjects with pterygium or 4.0% of the whole study population. Prevalence of pterygia increased from 6.7 ± 0.8% in the age group from 30-39 years, to 13.5 ± 1.2% in the age group from 50-59 years, to 25.3 ± 2.1% in the age group from 70-79 years. The prevalence of pterygia was associated with older age, male gender, lower level of education, lower body height, and (secondarily) with higher cylindrical refractive error. In a similar manner, the number of hours spent with vigorous outdoors activity was significantly associated with the prevalence of pterygia after adjustment for older age, male gender, lower body height and higher cylindrical refractive error. 

 The pterygium prevalence of 13% in our whole study population or of 15% (95% CI: 13.68, 16.01) in our population aged 40+ years was higher than the pterygium prevalence in recent other studies from India. In the South Indian Andhra Pradesh Eye Disease Study reported a pterygium prevalence of 11.7% [31]. In a study from the South Indian state of Tamil Nadu, pterygium prevalence was 9.5% [26]. Generally, a wide variation in the prevalence of pterygium has been reported ranging between 0.7% in Copenhagen [1], 1% in Kyoto, 2% in Greater Beijing in China [19], 2.8% in Victoria in Australia [5], 7.3% in the Blue Mountains Eye Study [3], 8.6% in Greenland [1], 7% in Singapore [6], 39% in rural Dali, China [23] and about 48% in Spain [21]. In a population-based study on rural Sumatra, Indonesia on 1210 adults aged 21+ years, pterygia were graded for severity and the basal and apical extent was measured by an ophthalmologist with a hand held slit lamp [8]. The age adjusted prevalence rate of any pterygium was 10.0% and of bilateral pterygia was 4.1%. Reasons for the marked differences in the reported prevalence of pterygia between the various studies may be differences in the study populations living in very different regions of the word, differences in the technological and socioeconomic developments, the lifestyle and living conditions of the populations examined, and in the study designs. To cite an example, populations may vary markedly in their exposure to ultraviolet light even if living in the same geographical region or latitude, if their socioeconomic background and lifestyle (mostly being indoors versus spending most of the time outdoors) varies profoundly. It may explain the relatively low prevalence of pterygium in Victoria, Australia despite the high number of sunny days per year. Another factor potentially influencing the prevalence of pterygia may be air pollution, what may be the reason for the relatively low pterygia prevalence of 2% in the region of Greater Beijing [11,19]. 

 Previous investigations have shown that a high number of factors were associated with pterygia suggesting a multi-factorial pathogenesis [4,18]. While the associations between pterygia and older age and outdoor occupations as a presumed surrogate of ultraviolet exposure appear to be unequivocal, the correlations with other parameters have remained uncertain [37-39]. The increase in the pterygium prevalence with older age may be due to the increased cumulative lifetime exposure to sunlight. Correspondingly, recent studies on the incidence of pterygium did not show an age-related dependence of the pterygium incidence [40]. 

 As in many previous studies [3,5,6,11,15,19,20,21,24,27,33], we also found an association between male gender and Pterygia. It is partially contradictory to some other studies such as the recent Andhra Pradesh Eye Disease Study and the study from Tamil Nadu [26,31], in which the prevalence of pterygia did not depend on gender. Interestingly, there were two studies from rural Dali in China and from Tibet, in which a higher pterygium prevalence among women as compared to men was found [13,23]. The different lifestyles and the differences in the division of labor between the genders may potentially be reasons for the discrepancies between the studies. 

 As in previous investigations [7,13,16,17,23,24,31], we also found an association between the pterygium prevalence and a lower level of education. With respect to associations with systemic diseases, we did not detect associations between pterygia and diabetes mellitus and arterial hypertension. It agrees with other studies form India and China [23,26,31], while in contrast, the Singapore Malay Eye Study detected an increased prevalence of pterygium among people with higher systolic blood pressure [20]. In a similar manner, neither alcohol consumption nor smoking was associated with pterygia in our study as it as in other previous investigations [21,23,26], while a study from North America reported on a protective effect of smoking [16]. In the Indian Andhra Pradesh Eye Disease Study, alcohol consumption showed a protective effect against pterygia when it was introduced into a multivariable model. The use of sunglasses as examined in some other studies [7,13,26] could not be addressed in our investigation since sunglasses were not in use in the study population. 

 Potential limitations of our study should be mentioned. First, as in any population-based study, selection bias could have accentuated some estimates and masked others. The overall participation rate in our survey was 80.1% what is comparable to other population-based studies. Second, our study was performed in a very rural location which bordered to so-called tribal regions in Central India. Our study population is therefore definitely not typical for the population of India as a whole. The peculiarity of our study was its study population for which the living conditions have not markedly changed within the last 100 years. Our study population may thus be considered as an extreme in the spectrum of study populations living at different levels of socioeconomic development. Third, our study as cross-section investigation did not allow statements on a longitudinal association between pterygia and age and other parameters. Finally, we did not apply any specific technology to quantify the actual ultraviolet exposure or life time ultraviolet exposure as used in previous studies [5,26]. Instead, we used outdoor activity as a surrogate measure of sunlight exposure. 

In conclusion, the prevalence of pterygium in rural Central India was about 13% among adult Indians aged 30+ years. Older age, lower educational level, male gender, lower body height and more time spent outdoors with vigorous work were associate factors. 
